# THE IHSS+ ADRD TRAINING PROJECT: BOLSTERING THE DEMENTIA CARE WORKFORCE IN CALIFORNIA

**DOI:** 10.1093/geroni/igac059.3069

**Published:** 2022-12-20

**Authors:** Jarmin Yeh, Brittney Pond, Matthew Beld, Corinne Eldridge, Leslie Ross

**Affiliations:** University of California, San Francisco, San Francisco, California, United States; University of California, San Francisco, San Francisco, California, United States; University of California, San Francisco, San Francisco, California, United States; Center for Caregiver Advancement, Los Angeles, California, United States; University of California, San Francisco, San Francisco, California, United States

## Abstract

California’s In-Home Supportive Services (IHSS) program provides vital home care to low-income consumers, some of whom live with Alzheimer’s disease or related dementias (ADRD). Yet, most IHSS caregivers receive little or no training in dementia care. With the prevalence of ADRD among Californians age 55+ projected to increase 127% by 2040, reaching over 1.5 million people, the IHSS consumer population living with ADRD will likely increase at a similar rate, exacerbating the need for dementia-trained home care workers. This poster describes preliminary outcomes of the IHSS+ ADRD Training Project, a 10-week, competency-based training program, aiming to reach 600 IHSS caregivers in Alameda County, California, by 2024. All planned in-person activities switched to virtual strategies due to the COVID-19 pandemic. To date, 348 IHSS caregivers have been trained through 16 classes, with 9 classes offered in English, 3 classes in Spanish, and 4 classes in Cantonese. A quasi-experimental, longitudinal design was used to evaluate the project’s impact through pre, post, and 3-month follow-up surveys. Caregiver outcome measures included the: 1) Dementia Knowledge Assessment Tool 2, 2) Fortinsky self-efficacy scale, 3) Caregiver Self-Assessment Questionnaire, and 4) Patient Health Questionnaire-2. Preliminary outcomes trend toward significant increases in caregivers’ knowledge about ADRD and self-efficacy to maximize care they provide to consumers. While caregivers also reported slight increases in stress and depression, they expressed high levels of satisfaction with the training. Future analysis will include comparing IHSS caregiver outcomes to healthcare utilization patterns of IHSS consumers before and after their caregiver’s participation in the training.

